# Aberrant Gray Matter Networks in Non-comorbid Medication-Naive Patients With Major Depressive Disorder and Those With Social Anxiety Disorder

**DOI:** 10.3389/fnhum.2020.00172

**Published:** 2020-06-10

**Authors:** Youjin Zhao, Running Niu, Du Lei, Chandan Shah, Yuan Xiao, Wenjing Zhang, Ziqi Chen, Su Lui, Qiyong Gong

**Affiliations:** ^1^Huaxi MR Research Center (HMRRC), Department of Radiology, West China Hospital of Sichuan University, Chengdu, China; ^2^Psychoradiology Research Unit of Chinese Academy of Medical Sciences, Functional and Molecular Imaging Key Laboratory of Sichuan Province, West China Hospital of Sichuan University, Chengdu, China; ^3^Department of Radiology, Sichuan Cancer Hospital and Institute, Sichuan Cancer Center, School of Medicine, University of Electronic Science and Technology of China, Chengdu, China

**Keywords:** major depressive disorder, social anxiety disorder, similarity-based gray matter network, graph theory, topological organization

## Abstract

Major depressive disorder (MDD) and social anxiety disorder (SAD) are among the most prevalent and frequently co-occurring psychiatric disorders in adults and may have, at least in part, a common etiology. However, the unique and the shared neuroanatomical characteristics of the two disorders have not been fully identified. The aim of this study was to compare the topological organization of gray matter networks between non-comorbid medication-naive MDD patients and SAD patients. High-resolution T1-weighted images were acquired from 37 non-comorbid medication-naive MDD patients, 24 non-comorbid medication-naive SAD patients, and 41 healthy controls. Single-subject gray matter graphs were extracted from structural MRI scans, and whole-brain neuroanatomic organization was compared across the three groups. The relationships between brain network measures and clinical characteristics were analyzed. Relative to healthy controls, both the MDD and the SAD patients showed global decreases in clustering coefficient, normalized clustering coefficient, and small-worldness and locally decreased nodal centralities and morphological connections in the left insular, lingual, and calcarine cortices. Compared with healthy controls, the SAD patients exhibited increased nodal centralities and morphological connections mainly involving the prefrontal cortex and the sensorimotor network. Furthermore, compared to the SAD patients, the MDD patients showed increased characteristic path length, reduced global efficiency, and decreased nodal centralities and morphological connections in the right middle occipital gyrus and the right postcentral gyrus. Our findings provide new evidence for shared and specific similarity-based gray matter network alterations in MDD and SAD and emphasize that the psychopathological changes in the right middle occipital gyrus and the right postcentral gyrus might be different between the two disorders.

## Introduction

Major depressive disorder (MDD) and social anxiety disorder (SAD) are among the most prevalent psychiatric disorders and are frequently comorbid with each other (Kessler et al., 2005b). The incidence of comorbidity between MDD and SAD ranges from 19.5 to 74.5% (Ohayon and Schatzberg, 2010; Koyuncu et al., 2014). From a clinical point of view, depression and anxiety share some symptoms, such as irritability (Vidal-Ribas et al., 2016), attention bias (Vidal-RibasSylvester et al., 2016), emotion dysregulation (Hofmann et al., 2012), and impaired social functioning (Saris et al., 2017). Furthermore, they also respond to the same treatment strategies (Ressler and Mayberg, 2007). In this line, it is plausible that depression and anxiety may have a similar etiology and pathophysiology based on common genetic polymorphisms (Mackinnon et al., 1990) and neurobiological vulnerability (Gulley and Nemeroff, 1993). However, the unique and the shared neuroanatomical characteristics of the two disorders have not been fully identified.

To date, few neuroimaging studies have directly compared the structural abnormalities between MDD and SAD. Our previous study found that MDD and SAD shared common patterns of gray matter (GM) abnormalities in the orbitofrontal–striatal–thalamic circuit, salience network, and dorsal attention network and that visual processing regions and the precentral cortex were disorder-specific for MDD and SAD, respectively (Zhao et al., 2017). Recent advances in brain connectomics have highlighted the disrupted brain networks in both MDD and SAD. Previous functional brain network studies have reported common global and local brain network property alterations in MDD and SAD, such as decreased global clustering coefficient (*C*_p_) (Luo et al., 2015; Zhu et al., 2017), increased global shortest path length (*L*_p_) (Luo et al., 2015; Zhu et al., 2017), and nodal centrality deficits in the posterior cingulate cortex (Zhu et al., 2017; Dong et al., 2019), insula (Li et al., 2017; Yang et al., 2017), and prefrontal cortex (Zhang et al., 2011; Yang et al., 2017). Furthermore, the MDD patients also manifested increased nodal efficiencies in the default mode network (Wang et al., 2017; Gong et al., 2018), and the SAD patients showed higher functional connectivity in the frontolimbic circuit (Yang et al., 2017).

Currently, functional MRI (fMRI) and diffusion tensor imaging (DTI) are the two most commonly used approaches to construct individual brain networks by estimating interregional functional connectivity (Biswal et al., 1995) or white matter connectivity (Iturria-Medina et al., 2007), respectively. Structural MRI (sMRI) is genetically heritable and is relatively insensitive to artifacts (e.g., head motion) when compared with fMRI/DTI (Alexander-Bloch et al., 2013); therefore, using brain GM anatomy to investigate brain networks in psychotic disorders may reveal more stable phenotypes related to altered anatomical organization (Zhang et al., 2020). The nodes in GM networks represent cortical areas that are considered to be connected when they covary in thickness or volume across subjects (Mechelli et al., 2005; He et al., 2007) or show structural similarity within a single subject (Tijms et al., 2012; Wang et al., 2016). However, networks of anatomical covariance have been calculated by creating only one network for a group of participants; thus, individual networks for each participant could not be examined and related to clinical parameters of interest. In contrast, the similarity-based GM network is constructed at the single-subject level rather than the group level, which provides an opportunity to examine associations of morphological network metrics with behavioral characteristics (Tijms et al., 2012).

To date, only one study has explored the similarity-based GM network in MDD patients (Chen et al., 2017), and none have done so in SAD patients. Chen et al. (2017) found lower global efficiency and higher modularity of the similarity-based GM network in MDD patients. However, they were unable to differentiate MDD from other related conditions, such as anxiety disorders. Therefore, we aimed to compare the global and local topological organizations of similarity-based GM networks between MDD patients and SAD patients. Since we previously demonstrated common and distinct GM volume and cortical thickness abnormalities in MDD patients and SAD patients (Zhao et al., 2017), we hypothesize that these patients would also manifest some common and distinct alterations in similarity-based GM networks and that these disruptions would be associated with the severity of the clinical symptoms. The study was conducted with non-comorbid medication-naive patients to reduce the impact of comorbidity and medications.

## Materials and Methods

### Participants

The study included 37 non-comorbid medication-naive MDD patients and 24 non-comorbid medication-naive SAD patients at the Mental Health Center, West China Hospital of Sichuan University. We included the subjects from our previous study (Zhao et al., 2017). The patients were consecutively recruited and diagnosed according to the Structured Clinical Interview for DSM-IV Axis I Disorders (SCID) (First et al., 1997). None of the patients had other psychiatric disorder comorbidity and had never received any psychotherapy or psychiatric medications before the MR examinations. All the MDD patients were evaluated using the Hamilton Anxiety Rating Scale (HAMA) and Hamilton Depression Rating Scale (HAMD). All the SAD patients were evaluated using the Liebowitz Social Anxiety Scale (LSAS).

In addition, 41 healthy controls (HCs) were recruited from the local area by poster advertisement and screened using the SCID non-patient edition to confirm the lifetime absence of psychiatric and neurological illness. They were interviewed to confirm that there was no family history of psychiatric illness.

The following exclusion criteria were applied to the three groups: (1) brain trauma, (2) neurological disorder, (3) alcohol or drug abuse, (4) pregnancy, (5) major physical illness such as cardiovascular disease or hepatitis, and (6) age less than 18 or over 60 years, as assessed by clinical evaluations and medical records. All the participants were right-handed as assessed with the Annett Handedness Scale (Annett, 1970). This study was approved by the ethics committee of West China Hospital of Sichuan University, and written informed consent was obtained from all the participants.

### MRI Acquisition and Imaging Preprocessing

The patients and comparison subjects underwent scanning using a 3-T MR scanner (Siemens Trio, Erlangen, Germany) with an eight-channel head coil. The head was stabilized with cushions and ear plugs. During scanning, the participants were instructed to relax their minds with their eyes closed without falling asleep. High-resolution T1-weighted images were acquired using a spoiled gradient recalled sequence with repetition time/echo time = 1,900/2.26 ms, flip angle = 9°, 176 sagittal slices with thickness = 1 mm, field of view = 240 × 240 mm^2^, and data matrix = 256 × 256, yielding an in-plane resolution of 0.94 × 0.94 mm^2^.

Structural images were preprocessed using Statistical Parametric Mapping 12 (SPM12) software^1^. Briefly, individual structural images were first segmented into GM, white matter, and cerebrospinal fluid using the unified segmentation model (Ashburner and Friston, 2005). Then, the images were spatially normalized to Montreal Neurological Institute coordinate space using Diffeomorphic Anatomical Registration Through Exponential Lie Algebra (Ashburner, 2007) and further non-linearly modulated to compensate for spatial normalization effects. The non-linear modulation essentially corrected for individual differences in brain size. Finally, the GM data were resampled to 2 × 2 × 2 mm^3^ voxels and spatially smoothed (Gaussian kernel with a full-width at half-maximum of 6 mm).

### Extraction of Brain Networks

Similarity-based GM networks were obtained based on intracortical similarity using a completely automated and data-driven method that has been described elsewhere (Tijms et al., 2012). Briefly, the method defined the network’s nodes as small regions of interest corresponding to 3 × 3 × 3 voxel cubes by dividing the GM. These cubes kept the 3D structure of the cortex intact, thereby using spatial information from the MRI scan in addition to the voxel values. Then, the structural similarity between two cubes was quantified by correlation coefficients. Next, the similarity matrices were binarized based on the significance of correlations after determining a threshold for each individual graph with a permutation-based method to ensure a < 5% (*SD* = 0.002) rate of spurious correlations between cubes. Only the positive similarity values survived this threshold. For a detailed description, refer to the work of Tijms et al. (2012).

Similarity-based GM networks defined in this way have different sizes. Since network properties can vary with network size (van Wijk et al., 2010), it is critical to have the same number of nodes and node sizes across participants by normalizing the GM networks. Therefore, we followed the methodology proposed by Batalle et al. (2013) to normalize single-subject GM networks based on the unified automated anatomical labeling (AAL) parcelation template. An AAL node was defined as the AAL region to which most voxels of each cube belong to. Each pair of AAL nodes was considered to be connected with a weight reflecting the strength of connection corresponding to the ratio of actual significant correlations divided by the total possible connections among cubes in pairs of nodes. The weight obtained is bounded between 0 and 1. Self-connections were excluded. This procedure resulted in a 90 × 90 weighted normalized network for each subject. One should note that the term “connection” in the present study refers to brain network edge indicating the statistically similar GM morphology of two cubes, which can exist in the absence of axonal connectivity.

### Network Properties

The network was constructed using GRETNA (v2.0.0) (Wang et al., 2015)^2^ as in previous brain network studies (Zhang et al., 2011, 2020). The upper and the lower limit of sparsity (*S*) threshold values used were determined to ensure that the thresholded networks were estimable for the small-worldness scalar and that the small-world index was larger than 1.0 (Watts and Strogatz, 1998). Our threshold range was 0.10 < *S* < 0.34, with an interval of 0.01. The area under the curve (AUC) was calculated for each network metric, providing a summarized scalar for the topological characterization of brain networks to avoid using an arbitrary single threshold selection (Zhang et al., 2011).

Both global and nodal network properties were calculated at each sparsity threshold. The following global metrics of small-world parameters (Watts and Strogatz, 1998) were examined: clustering coefficient (*C*_p_), characteristic path length (*L*_p_), normalized clustering coefficient (γ), normalized characteristic path length (λ), and small-worldness (σ). Network efficiency parameters, including local efficiency (*E*_loc_) and global efficiency (*E*_glob_) (Latora and Marchiori, 2001), were examined. Locally, nodal degree (κ) and nodal efficiency (*e*) were also examined in each AAL region. The detailed formulas, usages, and explanations of each parameter can be found in a previous study (Wang et al., 2011).

To determine whether the morphological brain networks were non-randomly organized, all the global network measures were separately normalized by the corresponding mean of 100 matched random networks. The random networks were generated using a topological rewiring algorithm (Maslov and Sneppen, 2002) that preserved the same number of nodes and edges and the same degree distribution as the real brain networks. Typically, a small-world network meets the conditions of γ = *C*_p__/_*C*_random_ > 1 and λ = *L*_p__/_*L*_random_ ≈ 1 (Watts and Strogatz, 1998); therefore, the small-world scalar σ = λ/γ is larger than 1 (Humphries et al., 2006).

### Statistical Analysis

The group differences in the AUCs of all of the network metrics (network efficiency, small-world properties, and nodal centrality measures) were compared by one-way analysis of variance (ANOVA) using GRETNA followed by *post hoc* tests using SPSS software^3^, version 22.0. For nodal centrality measures, Bonferroni corrections were applied in ANOVA and *post hoc* tests for multiple comparisons of *p* < 0.05.

Alterations in regional nodal metrics indicate an alteration in similarity with other nodes, as defined by structural correlation coefficients. In a secondary analysis, we thus compared the network correlation matrix (Fisher’s *z*-transformed) of aberrant nodes between the MDD patients, the SAD patients, and the healthy controls to identify the specific GM correlation alterations associated with nodes with altered metrics with the network-based statistics (NBS) method (Zalesky et al., 2010a). First, the nodes that exhibited significant intergroup differences in at least one of the nodal centralities (nodal degree and nodal efficiency) were chosen. Then, a subset of connection matrices connecting these altered nodes was created for each participant. Finally, the NBS approach was applied to define a set of suprathreshold links that connected with the abnormal nodes (*p* < 0.05, FWE-corrected network level). The threshold *t*-value was set as 3.1, and the number of permutations was 5,000. For a detailed description, see the work of Zalesky et al. (2010a). Brain networks were visualized with the BrainNet Viewer^4^.

After significant between-group differences were identified in the network metrics, we further assessed the relationships between altered network metrics and the illness duration and symptom severity scores (HAMA score and HAMD score for MDD patients; fear factor score, avoidance factor score, and LSAS total score for SAD patients) in the two patient groups. These assessments were performed by using partial correlations with age, gender, and education as covariates using SPSS software. The statistical analysis of the demographic and the clinical data was also performed with SPSS.

## Results

### Demographic and Clinical Data

We enrolled 37 MDD patients, 24 SAD patients, and 41 HCs (Table 1). No significant differences in age, gender, education, or handedness were found among the three groups. The SAD patient group showed longer illness durations than the MDD patient group, which might be attributable to the fact that the median age of onset for anxiety disorders is much earlier (11 years of age) than that for mood disorders (30 years of age) (Kessler et al., 2005a). However, no significant correlation was found between illness duration and any network metric in the two patient groups.

**TABLE 1:** Sample characteristics.

Characteristic	MDD (*n* = 37)	SAD (*n* = 24)	HCs (*n* = 41)	*p*-value
Age (year)	26.7 ± 7.1 (18–43)	24.5 ± 4.0 (18–32)	27.1 ± 7.2 (18–50)	0.113^†^
Sex, male/female	25/12	15/9	26/15	0.899^‡^
Education (year)	13.4 ± 3.0 (7–19.5)	14.0 ± 3.5 (8–21)	13.3 ± 2.6 (5–17)	0.860^†^
Duration (year)	2.0 ± 0.5 (0.6–3.0)	7.6 ± 3.8 (1.0–16.0)	–	0.000^§^
HAMA	28.1 ± 8.8 (12–43)	–	–	–
HAMD	25.0 ± 5.2 (16–36)	–	–	–
LSAS				
Fear factor	–	28.7 ± 12.5 (13–57)	–	–
Avoidance factor	–	28.4 ± 14.6 (4–58)	–	–
Total scale	–	57.0 ± 25.5 (23–115)	–	–

*Values are means ± standard deviations (minimum–maximum). ^†^*p*-values obtained by ANOVA model. ^‡^*p*-value obtained by two-tailed Pearson chi-square test. § *p*-value obtained by two-sample *t*-test. HAMA, Hamilton Anxiety Rating Scale; HAMD, Hamilton Depression Rating Scale; HCs, healthy controls; LSAS, Liebowitz Social Anxiety Scale; MDD, major depressive disorder; SAD, social anxiety disorder.*

### Alterations in Global Brain Network Properties

**FIGURE 1:**
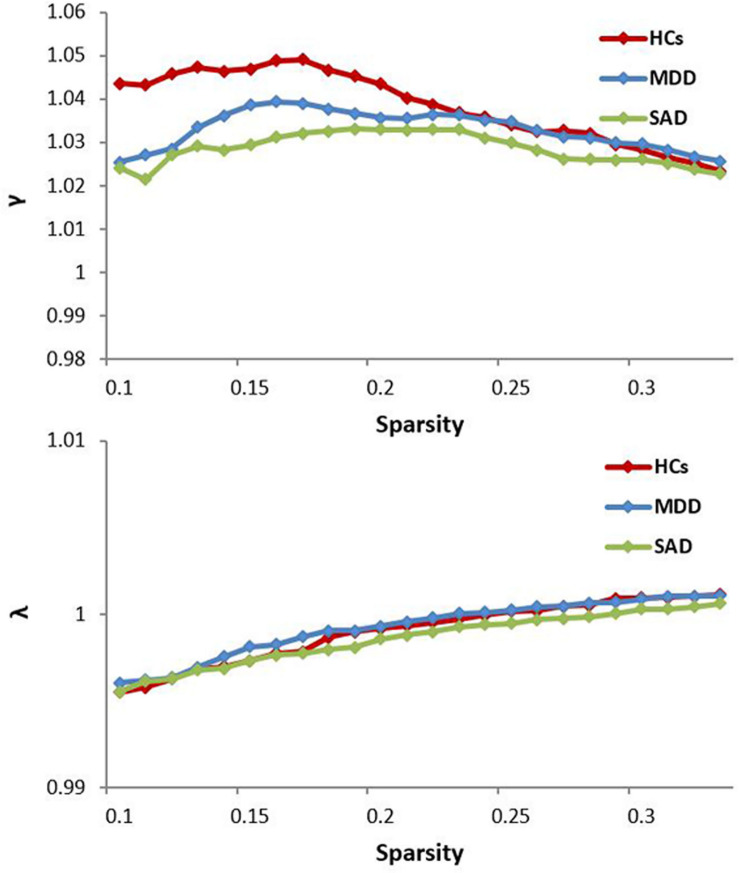
The key small-world parameters of the structural connectome as a function of the sparsity threshold. All MDD, SAD, and HCs groups showed a normalized clustering coefficient (γ) greater than 1 and a normalized characteristic path length (λ) approximately equal to 1, indicating that all groups exhibited a small-world topology (γ/λ > 1). HCs, healthy controls; MDD, major depressive disorder; SAD, social anxiety disorder.

The normalized GM graphs for each participant had a higher average clustering coefficient (γ > 1) than and similar characteristic path length (λ ≈ 1) to random reference networks, indicating that the three groups showed small-world topology in the brain functional connectome (γ/λ > 1) (Figure 1). The ANOVA results revealed significant differences in global efficiency, clustering coefficient, characteristic path length, normalized clustering coefficient, and small-worldness among the three groups (Table 2).

**TABLE 2:** One-way ANOVA and *post hoc* analyses results of the small-world properties and network efficiencies.

Network properties	ANOVA		MDD *vs.* SAD		MDD *vs.* HCs		SAD *vs.* HCs	
				
	*F*	*p*	*t*	*p*	*t*	*p*	*T*	*p*
*E*_glob_	4.71408	**0.01108**	−3.03047	**0.00312**	1.81845	0.07202	−1.48602	0.14045
*E*_loc_	0.07691	0.92603	−0.36755	0.71399	0.04529	0.96397	−0.33486	0.73844
*C*_p_	2.99004	**0.05485**	0.33410	0.73901	2.00557	**0.04763**	2.11012	**0.03737**
*L*_p_	3.93696	**0.02264**	2.79173	**0.00629**	−1.01741	0.31144	1.94929	0.05409
Gamma, γ	5.40580	**0.00591**	1.23289	0.22054	2.15410	**0.03366**	3.15773	**0.00211**
Lambda, λ	1.59539	0.20800	1.70758	0.09085	−1.24354	0.21660	0.64420	0.52093
Sigma, σ	5.34169	**0.00627**	1.01762	0.31134	2.30355	**0.02334**	3.07006	**0.00276**

*Significant group differences of network property (*p* < 0.05) are shown in bold font. C_*p*,_ clustering coefficient; *E*_*glob*,_ global efficiency; *E*_*loc*_, local efficiency; HCs, healthy controls; *L*_*p*,_ characteristic path length; MDD, major depressive disorder; γ, normalized clustering coefficient; λ, normalized characteristic path length; σ, small-worldness; SAD, social anxiety disorder.*

#### Shared Global Alterations

The *post hoc* analyses showed that both the MDD and the SAD groups, compared with the HC group, showed decreased clustering coefficient, normalized clustering coefficient, and small-worldness.

#### Global Alterations Between the Two Patient Groups

**FIGURE 2:**
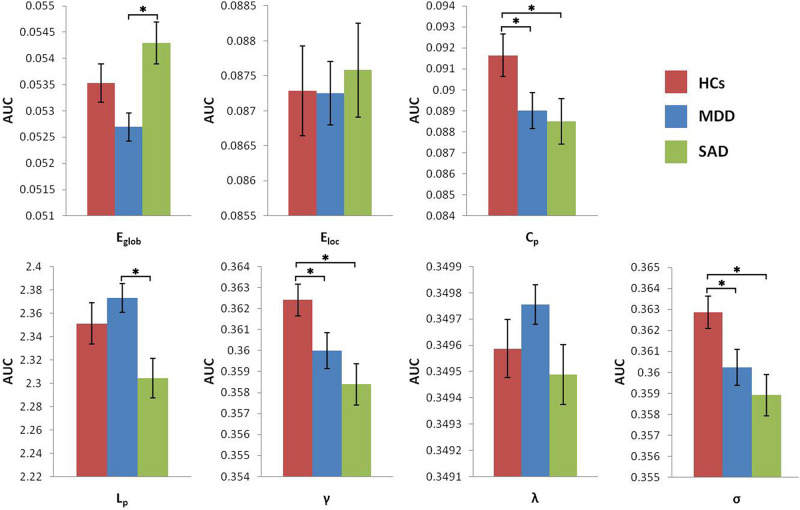
Graphs show differences in global topological properties among the three groups. ‘^∗^’ indicates a statistically significant difference between the two groups (*p* < 0.05). Error bars denote standard errors. AUC, area under the curve; C_p,_ clustering coefficient; E_glob,_ global efficiency; E_loc_, local efficiency; HCs, healthy controls; L_p,_ characteristic path length; MDD, major depressive disorder; γ, normalized clustering coefficient; λ, normalized characteristic path length; σ, small-worldness; SAD, social anxiety disorder.

The MDD group showed increased characteristic path length and decreased global efficiency compared with the SAD group (Figure 2).

### Alterations in Nodal Brain Network Properties

The ANOVA results identified 13 brain regions that show altered nodal centralities in at least one nodal metric among the three groups (Table 3) (*p* < 0.05, Bonferroni-corrected).

**TABLE 3:** Regions showing the altered nodal centralities among the three groups.

Brain areas	Nodal degree		Nodal efficiency	
		
	*F*	*p*	*F*	*p*
PreCG.L	0.00000	0.00460	8.98439	**0.00027**
IFG triang.L	8.21945	**0.00051**	0.00000	0.00087
SMA.R	0.00000	0.00078	11.71757	**0.00003**
OLF.L	0.00000	0.00157	8.35947	**0.00045**
REC.L	0.00000	0.00436	8.89678	**0.00029**
INS.L	22.76979	**0.00000**	19.45486	**0.00000**
CAL.L	24.95522	**0.00000**	22.93088	**0.00000**
LING.L	17.27615	**0.00000**	11.24193	**0.00004**
MOG.R	8.34149	**0.00046**	9.22267	**0.00022**
PoCG.L	0.00000	0.00096	8.14267	**0.00054**
PoCG.R	9.47064	**0.00018**	11.39219	**0.00004**
ITG.L	10.70796	**0.00006**	8.89667	**0.00029**
ITG.R	11.70368	**0.00003**	0.00000	0.00172

*Regions that survived Bonferroni correction for multiple comparisons (*p* < 0.05) in at least one of the nodal centralities (shown in bold font). CAL, calcarine cortex; IFG triang, inferior frontal gyrus triangular part; INS, insula; ITG, inferior temporal gyrus; L, left; LING, lingual gyrus; MOG, middle occipital gyrus; OLF, olfactory cortex; PoCG, postcentral gyrus; PreCG, precentral gyrus; R, right; REC, rectus gyrus; SMA, supplementary motor area.*

#### Shared Nodal Alterations

The *post hoc* analyses showed that both the MDD and the SAD patients, relative to the HCs, exhibited increased nodal centralities in the right supplementary motor area and decreased nodal centralities in the left insula, left calcarine cortex, and left lingual gyrus.

#### Specific Nodal Alterations

Compared with the HCs, the MDD patients showed decreased nodal centralities in the left olfactory cortex. Compared with the HCs, the SAD patients exhibited increased nodal centralities in the left precentral gyrus, the left inferior frontal gyrus triangular part, the left rectus gyrus, the right middle occipital gyrus, and the bilateral postcentral gyri and decreased nodal centralities in the bilateral inferior temporal gyri.

#### Nodal Alterations Between the Two Patient Groups

**FIGURE 3:**
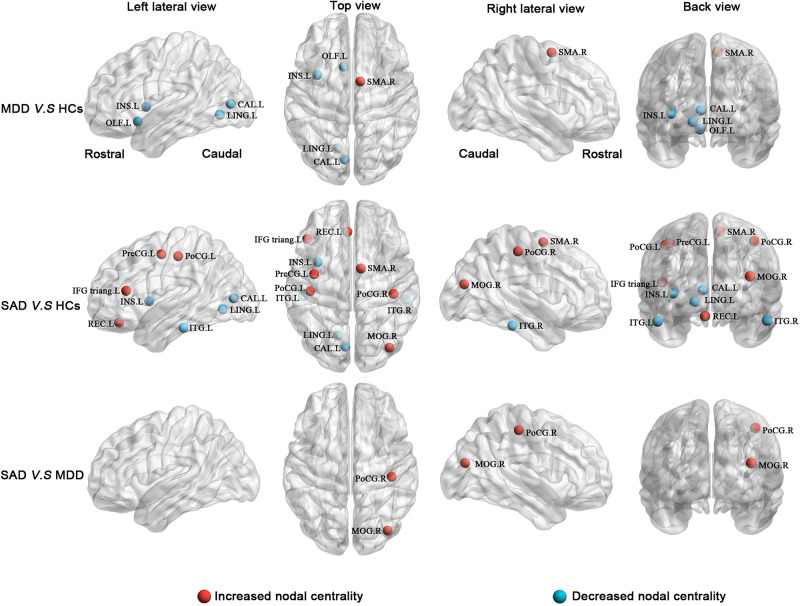
Between-group results of altered nodal centralities (corrected *p* < 0.05). **Row 1**: MDD patients compared with HCs. **Row 2**: SAD patients compared with HCs. **Row 3**: SAD patients compared with MDD patients. Increased nodal centralities are presented in red, and decreased nodal centralities are presented in blue. CAL, calcarine cortex; HCs, healthy controls; IFG triang, inferior frontal gyrus triangular part; INS, insula; ITG, inferior temporal gyrus; L, left; LING, lingual gyrus; MDD, major depressive disorder; MOG, middle occipital gyrus; OLF, olfactory cortex; PoCG, postcentral gyrus; PreCG, precentral gyrus; R, right; REC, rectus gyrus; SAD, social anxiety disorder; SMA, supplementary motor area.

Compared with the MDD patients, the SAD patients showed increased nodal centralities in the right middle occipital gyrus and the right postcentral gyrus (Figure 3 and Table 4).

**TABLE 4:** *Post hoc* analyses results of the altered nodal centralities among the three groups.

Brain areas	Nodal degree		Nodal efficiency	
		
	*t*	*p*	*t*	*p*
MDD > HCs				
SMA.R	3.66761	**0.00040**	3.82069	**0.00023**
MDD < HCs				
OLF.L	NS	NS	–4.09095	**0.00009**
INS.L	–4.01499	**0.00012**	–3.90977	**0.00017**
CAL.L	–6.31253	**0.00000**	–6.35749	**0.00000**
LING.L	–4.62736	**0.00001**	–3.73501	**0.00031**
SAD > HCs				
PreCG.L	NS	NS	4.54213	**0.00002**
IFG triang.L	3.82227	**0.00023**	3.95861	**0.00014**
SMA.R	NS	NS	4.31631	**0.00004**
REC.L	NS	NS	4.29987	**0.00004**
MOG.R	4.18331	**0.00006**	3.94715	**0.00015**
PoCG.L	4.10024	**0.00008**	4.21868	**0.00005**
PoCG.R	4.38365	**0.00003**	4.96416	**0.00000**
SAD < HCs				
INS.L	–6.38278	**0.00000**	–5.68477	**0.00000**
CAL.L	–5.85483	**0.00000**	–5.27233	**0.00000**
LING.L	–5.33300	**0.00000**	–3.90601	**0.00017**
ITG.L	–4.47191	**0.00002**	NS	NS
ITG.R	–4.70855	**0.00001**	NS	NS
SAD > MDD				
MOG.R	–3.32030	0.00126	–4.10445	**0.00008**
PoCG.R	–3.97494	**0.00013**	–3.85934	**0.00020**

*Regions that survived Bonferroni correction for multiple comparisons (*p* < 0.05) in at least one of the nodal centralities (shown in bold font). CAL, calcarine cortex; HCs, healthy controls; IFG triang, inferior frontal gyrus triangular part; INS, insula; ITG, inferior temporal gyrus; L, left; LING, lingual gyrus; MDD, major depressive disorder; MOG, middle occipital gyrus; NS, ANOVA not significant; OLF, olfactory cortex; PoCG, postcentral gyrus; PreCG, precentral gyrus; R, right; REC, rectus gyrus; SAD, social anxiety disorder; SMA, supplementary motor area.*

### Alterations in Morphological Connections

**FIGURE 4:**
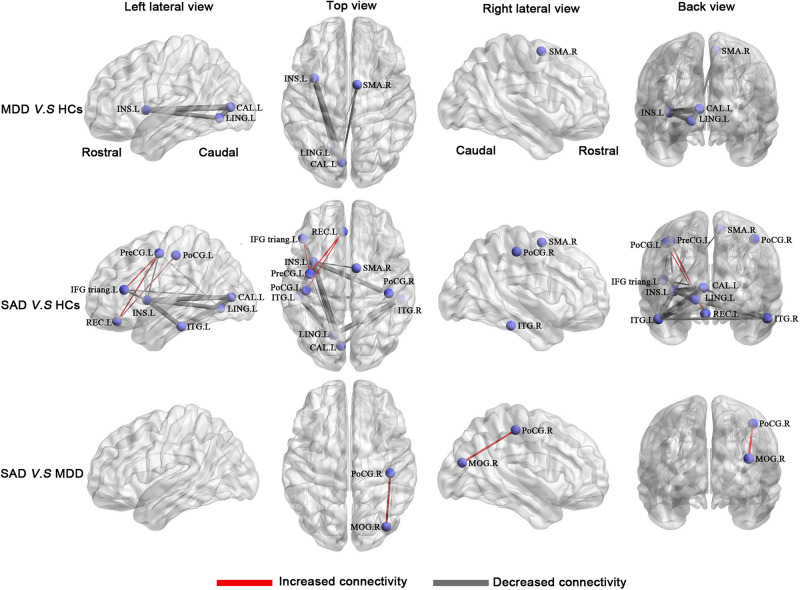
Between-group results of the brain structural connectome (corrected *p* < 0.05). **Row** 1: MDD patients compared with HCs. **Row 2**: SAD patients compared with HCs. **Row 3**: SAD patients compared with MDD patients. The purple nodes represent the regions with significantly altered morphological connections. The line width represents the *t*-value between groups, and the wider of the lines, the higher of the *t* values (for details of *t* values, see Table 5). Increased connections are presented in red, and decreased connections are presented in gray. Abbreviations: CAL, calcarine cortex; HCs, healthy controls; IFG triang, inferior frontal gyrus triangular part; INS, insula; ITG, inferior temporal gyrus; L, left; LING, lingual gyrus; MDD, major depressive disorder; MOG, middle occipital gyrus; PoCG, postcentral gyrus; PreCG, precentral gyrus; R, right; REC, rectus gyrus; SAD, social anxiety disorder; SMA, supplementary motor area.

Compared with the HCs, the MDD patients had a network with four nodes and five connections that were significantly altered, in which all connection alterations were decreased in the MDD group (corrected for multiple comparisons). Compared with the HCs, the SAD patients had a network with 11 nodes and 19 connections that were significantly altered, among which 16 connections were decreased and 3 connections were increased (corrected for multiple comparisons). Compared with the MDD patients, the SAD patients had a network with two nodes and one connection that was significantly altered (corrected for multiple comparisons). The networks involved brain regions in the frontal, the occipital, the temporal, and the parietal lobes (Figure 4 and Table 5).

**TABLE 5:** Disrupted morphological connections among the three groups.

Region 1	Category	Region 2	Category	*t*-score	Interlobe
**MDD < HCs**					
INS.L	Insula	CAL.L	Occipital lope	–7.37	Yes
CAL.L	Occipital lope	LING.L	Occipital lope	–6.53	No
INS.L	Insula	LING.L	Occipital lope	–6.35	Yes
SMA.R	Frontal lope	CAL.L	Occipital lope	–4.83	Yes
SMA.R	Frontal lope	LING.L	Occipital lope	–3.8	Yes
**SAD > HCs**					
PreCG.L	Frontal lope	REC.L	Frontal lope	4.47	No
REC.L	Frontal lope	PoCG.L	Parietal lope	4.14	Yes
PreCG.L	Frontal lope	IFGtriang.L	Frontal lope	3.91	No
**SAD < HCs**					
LING.L	Occipital lope	ITG.L	Temporal lope	–8.65	Yes
INS.L	Insula	ITG.R	Temporal lope	–7.80	Yes
LING.L	Occipital lope	ITG.R	Temporal lope	–7.73	Yes
INS.L	Insula	ITG.L	Temporal lope	–7.53	Yes
INS.L	Insula	CAL.L	Occipital lope	–7.49	Yes
INS.L	Insula	LING.L	Occipital lope	–7.08	Yes
CAL.L	Occipital lope	ITG.L	Temporal lope	–6.90	Yes
CAL.L	Occipital lope	LING.L	Occipital lope	–6.40	No
ITG.L	Temporal lope	ITG.R	Temporal lope	–5.34	No
IFG triang.L	Frontal lope	INS.L	Insula	–5.25	Yes
CAL.L	Occipital lope	ITG.R	Temporal lope	–5.07	Yes
PreCG.L	Frontal lope	INS.L	Insula	–4.61	Yes
IFG triang.L	Frontal lope	CAL.L	Occipital lope	–4.32	Yes
SMA.R	Frontal lope	INS.L	Insula	–4.26	Yes
PreCG.L	Frontal lope	LING.L	Occipital lope	–3.79	Yes
CAL.L	Occipital lope	PoCG.R	Parietal lope	–3.58	Yes
**SAD > MDD**					
MOG.R	Occipital lope	PoCG.R	Parietal lope	–4.06	Yes

*The connections are listed in descending order of statistical significance (*p* < 0.05). See Figure 3 for a graphical presentation of these connections. CAL, calcarine cortex; HCs, healthy controls; IFG triang, inferior frontal gyrus triangular part; INS, insula; ITG, inferior temporal gyrus; L, left; LING, lingual gyrus; MDD, major depressive disorder; PoCG, postcentral gyrus; PreCG, precentral gyrus; R, right; REC, rectus gyrus; SAD, social anxiety disorder; SMA, supplementary motor area.*

### Correlation of Network Alterations With Illness Duration and Symptom Severity

Using age, gender, and education as covariates, we did not detect significant correlations between network parameters and illness duration or symptom severity scores in the MDD patient group or in the SAD patient group (Table 6).

**TABLE 6:** *p*-values for the partial correlations of network alterations with illness duration and symptom severity in patients.

Network metrics		MDD			SAD			
			
		Illness duration	HAMA score	HAMD score	Illness duration	Fear factor score	Avoidance factor score	LSAS total score
**Global network metrics**								
	*E*_glob_	0.960	0.474	0.956	0.564	0.852	0.610	0.693
	*C*_p_	0.079	0.254	0.061	0.058	0.086	0.845	0.352
	*L*_p_	0.869	0.448	0.834	0.458	0.711	0.542	0.586
	Gamma, γ	0.322	0.802	0.450	0.209	0.863	0.861	0.984
	Sigma, σ	0.325	0.785	0.475	0.261	0.884	0.780	0.924
**Node degree**								
	IFG triang.L	0.913	0.816	0.707	0.448	0.627	0.236	0.347
	INS.L	0.629	0.548	0.875	0.483	0.713	0.445	0.526
	CAL.L	0.172	0.292	0.062	0.407	0.967	0.906	0.960
	LING.L	0.688	0.873	0.592	0.963	0.345	0.269	0.262
	MOG.R	0.177	0.952	0.703	0.603	0.659	0.139	0.277
	PoCG.L	0.511	0.892	0.998	0.559	0.159	0.987	0.495
	PoCG.R	0.232	0.067	0.837	0.294	0.860	0.762	0.925
	ITG.R	0.354	0.959	0.338	0.482	0.206	0.635	0.371
**Node efficiency**								
	PreCG.L	0.586	0.875	0.843	0.727	0.738	0.365	0.483
	SMA.R	0.132	0.602	0.107	0.990	0.267	0.324	0.258
	OLF.L	0.576	0.356	0.630	0.120	0.810	0.530	0.798
	REC.L	0.777	0.726	0.792	0.475	0.379	0.395	0.348
	INS.L	0.620	0.625	0.941	0.990	0.594	0.439	0.470
	CAL.L	0.268	0.617	0.096	0.595	0.660	0.613	0.606
	LING.L	0.642	0.459	0.310	0.930	0.071	0.055	0.065
	MOG.R	0.087	0.564	0.460	0.712	0.649	0.217	0.341
	PoCG.L	0.588	0.740	0.345	0.858	0.144	0.359	0.208
	PoCG.R	0.148	0.579	0.877	0.657	0.293	0.669	0.444
	ITG.L	0.215	0.062	0.904	0.256	0.805	0.553	0.636

*CAL, calcarine cortex; *C*_*p*_, clustering coefficient; *E*_*glob*_, global efficiency; HAMA, Hamilton Anxiety Rating Scale; HAMD, Hamilton Depression Rating Scale; IFG triang, inferior frontal gyrus triangular part; INS, insula; ITG, inferior temporal gyrus; L, left; LING, lingual gyrus; *L*_*p*_, characteristic path length; LSAS, Liebowitz Social Anxiety Scale; MDD, major depressive disorder; MOG, middle occipital gyrus; OLF, olfactory cortex; PoCG, postcentral gyrus; PreCG, precentral gyrus; R, right; REC, rectus gyrus; SAD, social anxiety disorder; SMA, supplementary motor area; γ, normalized clustering coefficient; σ, small-worldness.*

## Discussion

To the best of our knowledge, this is the first study to explore similarity-based, single-subject GM network abnormalities between non-comorbid medication-naive MDD patients and SAD patients. We found that both MDD patients and SAD patients exhibited a global decrease in clustering coefficient, normalized clustering coefficient, and small-worldness and locally decreased nodal centralities and morphological connections in the left insular, lingual, and calcarine cortices. Compared with the SAD group, the MDD group showed increased characteristic path length and reduced global efficiency and decreased nodal centralities and morphological connections in the right middle occipital gyrus and the postcentral gyrus. In addition, compared with the HCs, the SAD patients exhibited increased nodal centralities and morphological connections mainly involving the prefrontal cortex (i.e., bilateral postcentral gyri and left precentral gyrus) and sensorimotor network (i.e., left inferior frontal gyrus triangular part and left rectus gyrus). The present findings provide new evidence for GM network alterations at both the global and the local levels in MDD and in SAD patients without the impact of comorbidities and medications.

Both the MDD and the SAD patients, relative to the HCs, showed significantly decreased clustering coefficients and small-worldness in global network properties (Table 7). Segregation (reflected by clustering or local efficiency) and integration (reflected by path length and global efficiency) are two major organizational principles of human brain networks (Bullmore and Sporns, 2012). Our findings of a decreased clustering coefficient indicated a less specialized or segregated network organization, indexed by significantly lower clustering in the two patient groups. Previous studies have also reported a decreased global clustering coefficient of the network in MDD patients (Singh et al., 2013) and in SAD patients (Zhu et al., 2017). Although the brain networks of the patients and the controls showed “small-world” characteristics, the small-worldness was decreased in the two patient groups. The small-worldness reflects the balance between integration and segregation among all the nodes in the network. In our study, we did not find a significant increase in the normalized characteristic path length; therefore, the small-worldness reductions were predominantly due to reductions in the normalized clustering coefficients. Our results suggest a less optimized balance between global integration and local specialization in MDD patients and in SAD patients. In addition, compared to the SAD group, the MDD group showed increased characteristic path length and reduced global efficiency, which indicates reduced global integration of information processing and communication in MDD. Taken together, both the MDD patients and the SAD patients were characterized by a less segregated GM network organization, and compared to the SAD patients, the MDD patients showed reduced global integration.

**TABLE 7:** Brain network organization changes observed across different contrasts.

Contrast	*C*_p_	*E*_loc_	Segregation	*L*_p_	*E*_glob_	Integration	Σ
MDD vs. HCs	↓	–	↓	–	–	–	↓
SAD vs. HCs	↓	–	↓	–	–	–	↓
MDD vs. SAD	–	–	–	↑	↓	↓	–

*C_*p*,_ clustering coefficient; *E*_*glob*,_ global efficiency; *E*_*loc*_, local efficiency; HCs, healthy controls; *L*_*p*,_ characteristic path length; MDD, major depressive disorder; σ, small-worldness; SAD, social anxiety disorder.*

Compared with the HCs, both the MDD patients and the SAD patients showed decreased nodal centralities and morphological connections in the left insula and the occipital cortex (lingual and calcarine cortices). Nodal degree and nodal efficiency reflect the roles of nodes in information transmission and integration across the network (Sporns et al., 2007), so altered nodal centralities indicated changed regional function. The insula has been implicated in social function and awareness of bodily states (Adolfi et al., 2017), and the occipital cortex is involved in emotional facial processing, which is crucial for social functioning (Tao et al., 2013). The structural and functional abnormalities of these regions have also been reported in MDD (Wise et al., 2016; Zhao et al., 2017) and in SAD (Shang et al., 2014; Yang et al., 2017). However, the first similarity-based GM network study in MDD patients reported higher nodal efficiency in the insula and the calcarine cortex (Chen et al., 2017). These differences may be attributable to heterogeneities in the brain parcelation scheme and the clinical characteristics of the samples, such as illness duration, onset age, and the number of episodes. According to the axon tension theory, intracortical similarities could be due to the axonal connectivity that can influence the morphological measurements of the cortex (Van Essen, 1997; Hilgetag and Barbas, 2005). Indeed there are direct anatomical connections between the lingual and the calcarine cortices (Whittingstall et al., 2014) and between the insula and the occipital cortex (Jakab et al., 2012). Thus, the observed reduced morphological connections in the left insula and the occipital cortex may be due to neighboring white matter damage in MDD and in SAD; however, this is a speculative interpretation that requires direct testing.

The alterations in nodal centralities and morphological connections between the SAD patients and the HCs were more widespread than the differences observed between the MDD patients and the HCs. However, compared with the SAD group, the MDD group only showed significantly decreased nodal centralities and morphological connections in the right middle occipital gyrus and the postcentral gyrus. The middle occipital gyrus is involved in the perception of facial emotion (Fusar-Poli et al., 2009). The postcentral gyrus also plays an important role in emotional processing, including the identification of emotional significance in a stimulus, generation of emotional states, and regulation of emotion (Kropf et al., 2018). Studies have revealed increased resting-state and task-related activities in the occipital cortex in SAD, which might underlie the enhanced environmental scanning for potentially threatening or feared stimuli in SAD (Wang et al., 2018). Cortical thinning in the postcentral region was functionally related to the severity of social anxiety symptoms in SAD (Syal et al., 2012). Although we found no differences in nodal centralities in the right middle occipital gyrus and the postcentral gyrus between the MDD patients and the HCs, other researchers have reported reduced functions in the occipital lobe (Li et al., 2013) and the postcentral regions (Lai and Wu, 2015) in depressed patients. Furthermore, during a social evaluative threat task to assess the temporal aspects of the neural response to stress, participants diagnosed with social anxiety (SAD or SAD comorbid with MDD), relative to participants without diagnosed social anxiety (MDD or HCs), exhibited greater activation in the occipital cortex during instructions as well as less activation in the postcentral gyrus during recovery (Waugh et al., 2012). These data might indicate that the psychopathological changes in the two regions may be different between MDD and SAD patients, although this interpretation should proceed with caution because there is relatively little data on the neuroimaging differences between MDD and SAD.

### Limitations

There are several limitations to this study. First, the choice of network nodes has been somewhat arbitrary across published studies. We used the AAL atlas to parcellate the entire brain into 90 regions, which was the most commonly used method in previous studies. However, differences in template parcellations might have caused considerable variations in graph-based theoretical parameters, which must be explicitly compared in future work (Wang et al., 2009; Zalesky et al., 2010b). Second, although the similarity-based GM network method has been successfully applied to study neuropsychiatric disorders (Tijms et al., 2012; Chen et al., 2017; Niu et al., 2018), the biological significance of these network alterations has not been fully understood. Recent evidence suggests that intracortical similarities may arise from functional coherence, axonal connectivity, mutual trophic reinforcement, genetically mediated brain maturation, and experience-related plasticity (Alexander-Bloch et al., 2013; Evans, 2013). Third, because the two patient groups were previously recruited for two different projects, the MDD and the SAD patients were not evaluated with the same assessment scales. However, each patient was diagnosed with pure MDD or pure SAD by consensus case review according to the SCID. Fourth, the lack of an MDD/SAD comorbid group limits a complete description and delineation of our categorical model for MDD and SAD. Finally, our study was limited by the relatively small sample size; consequently, our preliminary results should be confirmed in a larger sample of patients and healthy controls in future studies.

## Conclusion

Using a similarity-based GM morphological network approach, we demonstrated that, globally, both the MDD and the SAD patients exhibited a less segregated GM network organization, while compared to the SAD patients, the MDD patients showed reduced global integration function; locally, both the MDD and the SAD patients demonstrated reduced nodal centralities and morphological connections in the left insula and occipital cortex (lingual and calcarine cortices), while compared with the SAD patients, the MDD patients showed decreased nodal centralities and morphological connections in the right middle occipital gyrus and the postcentral gyrus. Our findings provide new evidence for shared and specific similarity-based GM network alterations in MDD and SAD and emphasize that the psychopathological changes in the right middle occipital gyrus and the right postcentral gyrus might be different between the two disorders.

## Data Availability Statement

The datasets generated for this study are available on request to the corresponding author.

## Ethics Statement

The studies involving human participants were reviewed and approved by Ethics Committee of West China Hospital of Sichuan University. The patients/participants provided their written informed consent to participate in this study.

## Author Contributions

SL and QG developed the study design, provided the methodological advice, and supervised the conduct of the study. YZ and ZC collected the data. YZ, RN, and DL performed the data analysis. YZ, YX, and WZ generated the figures and tables. CS proofread the manuscript. YZ and SL wrote the manuscript, which all authors reviewed and approved for publication.

## Conflict of Interest

The authors declare that the research was conducted in the absence of any commercial or financial relationships that could be construed as a potential conflict of interest.

## Footnotes

1 http://www.fil.ion.ucl.ac.uk/spm/software

2 http://www.nitrc.org/projects/gretna/

3 http://www.spss.com

4 http://www.nitrc.org/projects/bnv/
